# Somatic characteristic, morphological asymmetry and postural stability of youth male canoeists compared to control. A cross-sectional study

**DOI:** 10.1371/journal.pone.0285997

**Published:** 2023-05-22

**Authors:** Magdalena Krzykała, Krzysztof Karpowicz, Małgorzata Karpowicz, Sylwia Bartkowiak, Anna Demuth, Urszula Czerniak, Jarosław Janowski

**Affiliations:** 1 Department of Methodology of Recreation, Poznan University of Physical Education, Poznań, Poland; 2 Department of Theory of Sports, Poznan University of Physical Education, Poznań, Poland; 3 Department of Team Sports Games, Poznan University of Physical Education, Poznań, Poland; 4 Department of Anthropology and Biometry, Poznan University of Physical Education, Poznań, Poland; Universiti Malaysia Terengganu, MALAYSIA

## Abstract

**Objective:**

To determine the relationships between selected morphological characteristics and the level of body composition asymmetry with postural stability among canoeists and a control group.

**Methods:**

The sample consisted of 43 males (21 canoeists 21.8±3.29 years and 22 university students 21.7±1.32 years). Measurements included body height and weight. Segmental body composition analysis was assessed using the bioelectrical impedance by determining: fat mass (FM, kg, %), fat-free mass (FFM, kg) and predicted muscle mass (PMM, kg). Postural stability was tested using the BIODEX Balance System. Anterior–posterior stability index (APSI), medial–lateral stability index (MLSI) and overall stability index (OSI) were calculated.

**Results:**

Our findings suggest that the canoeists had statistically lower level of fatty tissue compared to controls. There was a statistically important difference between groups in lower limb FM (% and kg). In both groups morphological asymmetry was observed, but in most cases—in athletes. Asymmetries between right and left arms appeared in all parameters, while asymmetries between right and left legs were noted in all parameters except FM (kg). There were relationships between stature and body weight with postural stability in canoeists. Canoeists demonstrated better balance than controls, particularly in the APSI. For all stability indices, significant differences were observed between right and left legs across all participants.

**Conclusions:**

Athletes, with larger asymmetries or poorer balance, require more attention to improve performance and reduce the risk of overload injury. Future studies needed to develop sport-specific level of morphofunctional asymmetry which would be optimal for sport results and health as well.

## Introduction

There are many factors that affect athletic performance like genetic potential, occupation, physical environment, daily nutrition, quality of sleep, training, injury coach-athlete relationship etc. [1]. Also, body structure and body composition (fat, muscle mass, total body water) are important determinants of performance in many sports [2]. Paddle sports have two sub-disciplines: canoeing and kayaking. There are a few differences between them. In a kayak, the paddler is seated and uses a double-bladed paddle pulling the blade through the water on alternate sides to move forward. In a canoe, on the other hand, the paddler kneels and uses a single-bladed paddle to propel the boat forward [3]. This has its consequences in the form of specific movement patterns. Canoeists athletes perform more asymmetrical moves. Canoeing has numerous requirements [4], including specific athlete anthropometric parameters [5, 6], which carry great importance. Hamano [7] reported on the significance of monitoring canoe athletes’ somatic characteristics and body composition during the process of planning training programmes and identifying athletes with a predisposition for this sport. Compared to kayak athletes, canoe athletes have smaller values for body height, sitting height, body weight, arm span, upper limb length and multiple body part circumferences [8, 9]. Ackland [9] showed that elite canoe sprinters have a more developed upper body, smaller buttocks and higher lean body mass values compared to beginner athletes and non-athletes. It has also been stated that appropriate shoulder and calf circumference sizes may be important in this sport [7]. Canoe athletes have been found to have 12%–18% body fat [10].

In athletes, there is a phenomenon of somatic adaptation to the specificity of a given discipline and its associated training loads, as athletes with different body structures can react to training loads in different ways. These discipline-specific loads are often reflected in the somatic parameters of athletes and are particularly noticeable in the case of unilateral sports. Many studies have shown that asymmetric sports disciplines lead to side-to-side differences in a variety of parameters [11–13]. One of them is canoeing. In classic canoeing—flatwater, competitors paddle in kayaks or in canoes. Whereas paddling in a kayak requires symmetric movements, paddlers in a canoe perform asymmetrical moves [14]. It is characterised by numerous one-sided activities, including the paddling technique (one-turn paddle) and the characteristic position of the athlete in the boat (half-kneeling in a lunge position), which can contribute to an asymmetric distribution of strength in certain muscle groups [15] and unbalanced morphological characteristics (muscle and fat). During paddling, the asymmetric tension of the lower limb musculature can cause asymmetries in body structure, particularly asymmetric muscle hypertrophy [16]. It can also lead to unbalanced forces on the boat.

Extreme specialisation (excessive practice of specific techniques) in sports such as fencing or canoeing can induce strong preferences for one side of the body and create asymmetric balance patterns [17, 18]. Also, in team games like hockey or handball asymmetrical positions are often assumed what could affects body posture and its stability [17, 19]. It is worth to know that some asymmetry level is also observed in population studies [20, 21].

Balance is fundamental motor skill providing the basis for the most movement task [22], particularly in its protective mechanism against injuries and is especially valid in the case of repetitive training over many years due to excessive practice of specific techniques. It can be deepened over time as a result of many years of unilateral training in sports such as canoeing, particularly as symmetrical training is generally not a focus in these sports [23, 24]. In many sports, though particularly in unilateral sports, athletes inevitably develop some degree of asymmetry; while, at a certain level, this is necessary to optimise performance, in excess, asymmetry can be unhealthy and inhibit performance. Balance is understood to be the ability to protect a given posture with minimal movement during fluctuations that are caused by static or dynamic conditions [25]. Static balance is the ability to maintain body position with minimal deviation while in a fixed position and on a stable surface [26], while dynamic balance is the ability to maintain body position while the body is moving or on an unstable surface [26, 27]. From a biomechanical point of view, balance is a state of the body in which the sum of the forces acting on it is equal to zero; the stabilising internal forces (motor effects of the nervous system) are equal to the destabilising external forces [28]. In canoeing, an incorrect movement or loss of balance can cause less effective work or even a fall overboard; balance is thus a critical component of this discipline [29].

Canoeing is one of the disciplines that involves the asymmetrical performance of motor activities. It also requires an above-average level of balance and nervous system efficiency due to the performance of starting activities on an unstable surface such as water. Estimating the level of asymmetry provides an opportunity to counteract unilateral overloading of the body, which can lead to injury in extreme cases. It was concluded in the literature that asymmetry of athletes is one of the main causes of musculoskeletal diseases and sports injuries [30]. This asymmetry could be caused f.e. by abnormality of the spine [31]. Moreover, asymmetry of bilateral loading can contribute to the increase of unilateral limb damage and injuries [32]. In asymmetry sport disciplines uneven development of muscle mass may cause disorders of posture, leading to negative changes in the skeletal system [33]. It was also emphasizing that lower limbs muscle strength asymmetry is associated with a high risk of injury [34, 35].

The true is however that there are inconsistent findings about eventual association between bilateral lower limb asymmetries and injury occurrence, even less is known about upper limb asymmetries (which would be important, especially in highly asymmetric sports such as canoeing) and injury occurrence. That is why the reduction of injury occurrence may be achieved through the identification of risk factors, which are specific for certain sport, also connected with morpho-functional body asymmetries what could be helpful for coaches.

In the context of the above issues, the main purpose of this study was to determine the relationships between selected morphological characteristics and the level of body composition asymmetry with postural stability among canoeists and a control group.

## Material and methods

The study group consisted of 21 male canoeists aged 21.8±2.29 years from the Wielkopolska region of Poland; all participants had 1.5 to 5 years of practice national or international level. The studies were carried out taking into account the training periodization, at the beginning of the preparation period. The control group was made up of 22 university students aged 21.7±1.32 years who were engaged in recreational sports activities but were not part of any competitive sports training.

All participants received clear information regarding the study, including the risks and benefits of participation, and provided written informed consent. The study was conducted according to the latest version of the Declaration of Helsinki, and the local ethics board of Poznan University of Medical Sciences approved the study protocol (approval number: 172/16).

### Study protocol

Before the measurements all participants completed a questionnaire. Prior to beginning the research, all participants declared their hand preference (which hand they write with) [36].

The next step was anthropometry protocol as well as body composition measurement. The last stage of research was postural stability assessment.

### Anthropometric measurements and body composition analysis

Anthropometric measurements were performed by an experienced researcher following the recommendations of Martin and Saller [37]. Body height was measured by stadiometer (GPM, Switzerland), to the nearest 1 mm.

Body weight and segmental body composition were determined using the Tanita analyser (BIA; Tanita MC-780MA analyser, Japan). Following parameters were assessed: fat mass by percentage and in kilograms (FM% and FMkg), fat-free mass in kilograms (FFMkg) and muscle mass in kilograms (PMMkg) for right and left upper and lower limbs, based on the directions and procedures of the manufacturer [38]. All subjects were asked to refrain from drinking or eating, empty their bladders, wear light clothing and remove metal objects before the examination [39]. During the test, participants stood erect with their bare feet on the contact electrodes while holding the electrodes of the BIA unit in their hands. All assessments were completed in a single visit scheduled in the morning.

For each subject, all measurements (anthropometric and BIA measurements) were taken on the same day in an indoor environment, where the temperature was maintained at a constant 22°C.

### Postural stability measurement

All participants underwent postural stability testing on the BIODEX Balance System SD (BBS; Biodex, Shirley, NY, USA) by the same well-trained researcher. A stabilographic platform was employed, which enables the measurement of changes in the forces acting on the ground in the anterior–posterior or lateral directions that affect the point of application of the resultant force, which is referred to as the centre of pressure (COP) [28]. The platform is an instrument designed to measure and train postural stability on fixed or unstable surfaces. The BBS system consists of a circular platform that can move freely and simultaneously in the front–rear and centre–lateral axes and has a display that provides real-time feedback on the subject’s posture.

Participants stood on the BBS platform in double-leg and single-leg (right and left leg) positions, keeping their eyes open and looking at the screen throughout all measurement trials. All trials were conducted in athletic shoes, and foot position was recorded using coordinates on a platform grid and entered into the instrument computer. During the measurements, the subjects held their arms freely at their sides and were verbally instructed to stand as still as possible.

A protocol to determine postural stability (Postural Stability Test) with a standard software configuration was implemented on the platform. The protocol consisted of 3 trials of 20 seconds each with a 10-second interval between trials during which the platform was stationary. The interval between double-leg and right and left single-leg tests was 1 minute. The result was the average value of the tree values [40].

The stability indices anterior–posterior stability index (APSI), medial–lateral stability index (MLSI) and overall stability index (OSI; a combination of the APSI and MLSI that is sensitive to changes in both directions) showing the variance of the movement of the center of gravity expressed in degrees, were used for statistical analysis. All stability indices were determined in the following manner [41]:

MLSI = √ [Σ (0 − x)^2^/ [number of data points]
APSI = √ [Σ (0 − y)^2^/ number of data points]
OSI = √ [Σ (0-y)^2^ + Σ (0-x)^2^/ number of data points]

A score of zero represents complete stability, and the larger the deviation from zero, the larger the instability and postural sway [42], indicating difficulty maintaining balance. For each type of deviation, the standard variation of the individual indicators was also automatically provided by the device.

The results of the measurements were stored in the BBS stabilometer, and after the completion of the testing, the mean values of the balance indices were calculated. The athletes and controls were verbally familiarised with the assessment protocol and a 10-minute warm-up was performed by all participants prior to the initiation of the testing protocols.

### Data analysis

All parameters were reported as mean values and standard deviation. The normal distribution of the data was tested by the Shapiro–Wilk test and homoscedasticity was assessed by Levene’s test.

Descriptive characteristics were compared to explore possible differences between canoeists and controls in height, weight and stability indices. For parameters with normal distribution and homogeneity, Student’s t-test for independent sample (t) was used, and for non-normally distributed variables, the Mann-Whitney U (Z) test was used. Multivariate analysis of variance (multifactorial ANOVA leg x group) (F) was used to examine differences in the simultaneous effect of leg (right-left) and group (canoeists-controls) as well as leg-group interactions. Tukey’s post hoc procedure was used to identify specific statistically important differences in effects and interactions. The significance of differences in mean values between right and left body segments was checked with Student’s t-test for unpaired data for variables with normal distribution and the Wilcoxon test for variables with non-normal distribution (differences between the control group and canoeists as well as differences between the right and left side in the control group and in the group of canoeists separately).

Spearman’s rank correlation coefficient was used to determine the relationship between variables due to the no normal distribution for at least one variable. Statistical power of the test was checked with Cohen’s criteria, an effect size ≥ 0.20 and < 0.50 was considered small, ≥ 0.50 and < 0.80 was considered medium and ≥ 0.80 was considered large [9]. The effect size was selected taking into account the group-leg interaction.

Statistical significance was found at p<0.05, while the tendency to significance at p<0.10, p≥0.05 was considered statistically insignificant.

All analyses were performed using the Statistica 13.3 software program (TIBCO Software Inc., 2017).

## Results

### Somatic characteristics and body composition asymmetry

Descriptive statistics of the canoeists and control group are shown in Table 1. Both groups (canoe athletes and control) were homogeneous in terms of body height (t = 1.32, p = 0.194) and body weight (Z = 0.99, p = 0.320). The only statistically significant differences in mean somatic parameters between the two groups were in FM% (t = −2.71, p = 0.010, d = 0.85), which had a large effect size, and FMkg (t = −2.09, p = 0.043, d = 0.65), which had a medium effect size. Canoeists had lower FM% and FMkg compared to controls. Trends toward significance occurred for FFMkg (t = 1.90, p = 0.066, d = 0.58) and PMMkg (t = 1.99, p = 0.054, d = 0.62) with a medium effect size in both cases.

**Table 1:** Descriptive characteristics of morphological variables among canoeists and control group (M±SD).

Variables	Canoeists n = 21	Control n = 22	p value between groups
Body height [cm]	181.3 ± 6.42	178.9 ± 6.13	0.194^a^
Body mass [kg]	80.1 ± 10.50	78.1 ± 9.84	0.320^c^
FM [%]	13.1 ± 4.26	16.6 ± 4.00	0.010^a^*
FM [kg]	10.6 ± 3.56	13.3 ± 4.72	0.043^a^*
FFM [kg]	69.6 ± 9.62	64.9 ± 6.10	0.066^b^
PMM [kg]	66.4 ± 9.16	61.7 ± 5.81	0.054^b^

FM, fat mass; FFM, fat free mass; PMM, predicted muscle mass.

^a^Student’s t-test;

^b^ variance estimation test,

^c^ Mann-Whitney U test.

*Adopted level of significance p < 0.05.

Table 2 shows the site-specific body composition.

**Table 2:** Side-specific FM, FFM and PMM in canoeists and control (M±SD).

	Canoeists (n = 21)		Control (n = 22)		
Body segment	M ± SD	p value between sides	M ± SD	p value between sides	p value between groups
**FM [%]**					
Left arm	15.7 ± 4.41	0.032*	15.9 ± 3.16	<0.001*	0.860
Right arm	15.0 ± 3.74		15.1 ± 2.78		0.904
Left leg	16.7 ± 6.02	0.043*	13.4 ± 3.27	<0.001*	0.047*
Right leg	15.4 ± 4.91		12.7 ± 3.50		0.046*
**FM [kg]**					
Left arm	0.8 ± 0.18	0.029*	0.8 ± 0.23	0.017*	0.651
Right arm	0.7 ± 0.16		0.7 ± 0.22		0.890
Left leg	2.3 ± 0.81	0.144	1.8 ± 0.60	0.088	0.032*
Right leg	2.2 ± 0.71		1.8 ± 0.63		0.033*
**FFM [kg]**					
Left arm	4.1 ± 0.81	0.000*	3.9 ± 0.49	0.825	0.295
Right arm	4.0 ± 0.77		3.9 ± 0.47		0.615
Left leg	11.4 ± 1.48	0.000*	11.4 ± 1.05	<0.001*	0.848
Right leg	12.0 ± 1.37		11.8 ± 1.01		0.566
**PMM [kg]**					
Left arm	3.9 ± 0.77	0.000*	3.7 ± 0.45	0.825	0.348
Right arm	3.8 ± 0.72		3.7 ± 0.44		0.682
Left leg	10.9 ± 1.38	0.000*	10.8 ± 0.99	<0.001*	0.829
Right leg	11.3 ± 1.27		11.4 ± 0.95		0.594

FM, fat mass; FFM, fat free mass; PMM, predicted muscle mass.

*Adopted level of significance p < 0.05.

The analysis of FM% side-to-side differentiation indicated significantly higher values of this variable occurred in the left compared to right arm both in canoeists and control as well (p = 0.032 and p<0.001, respectively). Similar situation was observed in the case of legs (p = 0.043 and p = 0.001, respectively). When comparing asymmetry level of this parameter between groups, significant difference was noticed in the case of right legs (p = 0.046) and left legs (p = 0.047), but no in arms.

The absolute values for FMkg also showed that left segments were dominant in canoeists and control but the differences were statistically significant only in arms (p = 0.029 and p = 0.017, respectively). Such differences were not observed for the lower extremities. However, the significant differences were noticed between groups in the case of right legs (p = 0.033) and left legs (p = 0.032).

The analysis of FFMkg asymmetry revealed that in the canoeists significantly higher values of this variable occurred in left compared to right arm (p<0.001) and in left compared to right leg (p<0.001). In the control, the significant difference was noticed between left and right leg (p<0.001).

When analyzing PMMkg value it was noticed, that there was significant differentiation in canoeists in the case of arms (p<0.001) and legs (p<0.001), whereas in the control group only in the case of legs (p<0.001).

There were no significant differences between left and right arms as well as legs in FFMkg and PMMkg between the canoeists and control.

### Postural stability

Table 3 presents the postural stability variables measured in all participants.

**Table 3:** Descriptive characteristics of functional variables (in degrees) according to measurement position among canoeists and control group (M±SD).

Variables	Measurement position		Canoeists n = 21	Control n = 22
OSI	Both legs		0.30 ± 0.13^c^	0.41 ± 0.27
APSI			0.22 ± 0.10^c^^#^	0.33 ± 0.16
MLSI			0.12 ± 0.07^c^	0.17 ± 0.16
OSI	One leg	Right	0.75 ± 0.18	0.69 ± 0.19^$^
		Left	0.81 ± 0.21^#^	1.01 ± 0.26
APSI	One leg*	Right	0.52 ± 0.15^#^	1.00 ± 0.35^$^
		Left	0.58 ± 0.14	0.58 ± 0.18
MLSI	One leg*	Right	0.41 ± 0.10	0.57 ± 0.24
		Left	0.47 ± 0.16	0.70 ± 0.23

OSI, overall stability index; APSI, anterior-posterior stability index; MLSI, medial-lateral stability index.

*Significant difference between groups p<0.05 (without limb division).

^#^Significant different from the control group p<0.05.

^$^Significant different from left limb p<0.05.

^a^Student’s t-test;

^b^Variance estimation test;

^c^Mann-Whitney U test.

There were no significant differences between left and right arms as well as legs in FFMkg and PMMkg between the canoeists and control.

In the case of all stability indices (OSI, APSI and MLSI), the canoeists demonstrated better balance scores than the controls (values closer to zero), though only APSI was significantly different between groups (t = −2.46, p = 0.018, d = 0.82).

When comparing postural indices without separating single-leg positions between the canoeists and controls, the groups were significantly different from each other and there was an interaction between groups in APSI (F = 21.18, p ≤ 0.0001, d = 1.02) and MLSI (F = 12.77, p = 0.001, d = 0.79).

Statistically significant effects of groups and an interaction of measurement condition were observed for OSI (F = 28.59, p ≤ 0.000). This was influence by a statistically significant difference between the groups in the left leg (p = 0.017, d = 1.16). In the right leg there were no differences between the two groups (p = 0.778).

For APSI, the same observation was made for the right single-leg position (F = 28.84, p = 0.000, d = 1.78). In the control group, the right single-leg position was significantly different than the left single-leg position in OSI (F = 28.59, p = 0.000, d = 1.69) and APSI (F = 28.84, p = 0.000, d = 1.70).

### Correlations between somatic characteristics, body composition asymmetry and stability indexes

Taking into consideration basic anthropometric parameters in relation to postural stability it was found, that in canoe athletes, only body height correlated with APSI (r = −0.43); lower body height values correlated with poorer balance scores (Table 4).

**Table 4:** Spearman rank correlations between stability indexes (in degrees) and basic somatic characteristics in canoeists and control group.

Stability indices	Measuremnt position	Height (cm)		Weight (kg)	
		Canoeists n = 21	Control n = 22	Canoeists n = 21	Control n = 22
		r p value			
OSI	Both legs	-0.21	-0.20	-0.17	0.14
		0.364	0.383	0.473	0.546
	Right leg	-0.34	-0.12	-0.49	-0.01
		0.132	0.591	0.026*	0.964
	Left leg	-0.43	-0.19	-0.45	-0.01
		0.054 ^#^	0.411	0.042*	0.975
APSI	Both legs	-0.43	-0.30	-0.30	0.10
		0.049*	0.181	0.186	0.546
	Right leg	-0.33	-0.20	-0.42	-0.13
		0.149	0.377	0.056^#^	0.561
	Left leg	-0.33	-0.11	-0.44	0.11
		0.143	0.636	0.044*	0.641
MLSI	Both legs	-0.20	-0.42	-0.13	-0.10
		0.397	0.055^#^	0.589	0.665
	Right leg	-0.15	-0.08	-0.22	-0.22
		0.516	0.726	0.330	0.347
	Left leg	-0.08	-0.24	-0.07	-0.01
		0.737	0.304	0.764	0.984

OSI, overall stability index; APSI, anterior-posterior stability index; MLSI—medial-lateral stability index.

*p<0.05 statistically significant value;

^#^p<0.01 tendency to statistical significance; p≥0.05 statistically insignificant value.

Next, side-to-side differences between single-leg stability indices and basic somatic parameters were evaluated. The canoe athlete group demonstrated a statistically significant correlation between body weight and OSI in the right single-leg position (r = −0.49) and left single-leg position (r = −0.45). In both the right and left lower limbs, there was a negative, statistically significant relationship between body weight and OSI such that the lower the body weight, the poorer the OSI result. There was also a negative, statistically significant relationship between body weight and APSI in the left single-leg position (p = 0.043), with athletes with lower body weight achieving poorer APSI results. In the right single-leg positions, a tendency toward significance was observed (p = 0.056). No significant correlations between body weight and single-leg stability indices were observed in the control group.

The next question was whether body composition asymmetry determines postural stability in canoeists and control. In the case of canoeists, only in the case of FMkg legs asymmetry correlation appeared with right leg OSI and right leg MLSI postural stability. Among control, FMkg legs asymmetry correlated with OSI whereas FM% legs asymmetry correlated with MLSI. For the remaining bias differences, no correlations were observed with any of the stability indexes.

## Discussion

The main objective of the present study was to assess the differentiation among some morphological characteristics with segmental analysis of body composition and postural stability among canoeists and control in order to determine whether athletes are more asymmetric in morphological and functional characteristics of the body than non-athletes. It’s seems to be important because previous observations demonstrated that certain somatic characteristics could affect athletes’ balance [43, 44] and that basic somatic and body composition variables should be taken into account when identifying the most talented canoeists [9, 45] and systematically monitored during canoe training programmes [7]. Robinson [46] showed that athletes’ anthropometric characteristics have a large impact on competitiveness in the canoe sprint.

In previous studies, elite canoe sprinters in Japan had a height of 171.2±5.50 cm and a body mass of 71.8±8.20 kg, while university canoe athletes (aged 20.6±0.9 years) in Hamano [7] study had a height of 172.8±5.20 cm and a body mass of 70.8±7.80 kg. Young Bulgarian canoe athletes aged 15.12±0.14 years had a height of 172.3±5.51 cm and weight of 66.1±7.29 kg [47]. Some authors have stated that satisfactory results at the international level can only be achieved by canoeists who are 180–190 cm in height [48, 49]. The athletes analysed in the present study fulfilled this characteristic, as the average height of the canoe athletes was 181.3±6.42 cm, making the athletes taller than the controls (178.9±6.13 cm). The canoe athletes also had higher mean body weight values than the controls (80.1±10.50 kg and 78.1±9.84 kg, respectively). The reported body height and mass of the junior and teenage Polish national canoeing team (aged 18.7 years; 176.9 cm height and 75.5 kg mass) were smaller than those found in our study [8].

Body height and weight are connected to balance. Subjects who are taller and heavier have been found to demonstrate worse balance than their shorter and lighter counterparts [50–52]. Hue [52] moreover stated, that body weight alone explained 52% of the variance of static balance in adults, and body weight has been reported to play an important role in balance maintenance in unstable conditions [5]. In the present study, athletes with lower body height demonstrated greater anterior–posterior sway as measured by the APSI. In addition, decreased body weight in the athlete group was connected with OSI in the right and left single-leg position as well as with APSI in the left single-leg position. Relationships between height and weight and postural stability were not observed in the control group.

Athletes had lower levels of body fat and higher levels of fat-free mass and muscle mass than controls, though the differences were small and statistically significant only for fat mass. Hagner-Derengowska [8] showed that canoe athletes had a small fatty tissue percentage and higher fat-free mass values compared to control. This may be attributed to the key goal of training in these athletes being improving strength and speed, which favours muscle tissue hypertrophy [53]. In addition, adipose tissue levels are important in canoeing because they directly affect the submersion of the boat, which in turn impacts the drag on the boat and thus the boat’s speed [43]. Moreover, additional weight (especially connected with excess adipose tissue) of the canoe athlete, affects the submersion of the boat, which in turn impacts its drag and thus changes the boat’s speed [33].

The canoe athletes in this study were found to have significant asymmetries in fat-free mass and muscle mass between the right and left arms as well as between the right and left legs.

Other authors have stated that long-term canoe paddling does not increase muscle mass asymmetries and that long-term training can actually help to equalise muscle mass in the lower limbs [33]. Also, in the case of fatty tissue (FM%) distribution in arms and legs significant differences were observed both in athletes and control. Kašček Bučinel [44] reported that body symmetry is a critical factor of postural stability. The greater body symmetry connected with less difference between the right and left sides, the better static balance [40]. Particularly among athletes, good postural balance reduces the risk of injuries [54, 55] and is an important component of physical performance. On the other hand, higher racing speed of canoeist has been associated with more pronounced asymmetry in lumbar spine flexion in the coronal plane due to prolonged training leading to adaptive changes in the body [24]. However, it remains unclear when asymmetry is acceptable and when it has negative health consequences; thus, further observations are needed.

When compared two groups in morphological asymmetry level it was observed that only in case of lower limb fat tissue distribution significant differentiation appeared.

Previous findings appear to show the opposite tendency regarding the effect of prolonged training on asymmetry, with lower levels of asymmetry in lower limb muscle mass observed in advanced canoe competitors compared to competitors at the beginning of their careers [56]. This may be due to effective general development training, which reduces asymmetries that may have occurred during biological development in young athletes. It is also possible that the training used in young athletes focuses largely on specialised activities, which can cause muscle mass asymmetries. It is well known that in many sports (especially unilateral ones), certain interlimb asymmetry is acceptable, so it should not be automatically associated with worse performance [57, 58]. According to Afonso and co-authors [59], some level of asymmetry seems to be treated as the norm rather than something special.

Arol and Eroğlu [29] stated that static and dynamic balance ability is dependent on the specific training methods employed. Standing balance is connected with the ability to adapt and readapt quickly and effectively to a given task and environment [47]. In canoe athletes specifically, balance is a valuable skill.

It should be noted that the canoe athletes in the present study had better balance than the controls. This is in line with the findings yielded by other studies focusing on sports where biomechanical stability is required to maintain balance during activity what is essential for safety and effective work [60]. Stambolieva [47] similarly reported significantly better body postural stability in young male canoe and kayak athletes compared to controls due to the balance requirements specific to these sports, which naturally improve athletes’ postural control. Afonso et al. suggest that those with larger asymmetries or lower level of balance, require more attention to improve performance and reduce the risk of overload injuries (bearing in mind that some degree of interlimb asymmetry is not always associated with injury occurrence) [59].

## Conclusion

Our findings suggest that the analysed canoeists had statistically lower level of fatty tissue compared to controls. In both groups morphological asymmetry was observed, but in most cases in athletes. For canoeists, side-to-side differentiation was observed for all parameters. The exception was FMkg in the lower extremities, which did not differ significantly. In the case of control symmetry was noticed between legs FMkg, arms FFMkg and arms PPMkg. Statistically significant differences between sides between both groups appeared only in legs FM (%, kg).

The present analysis showed that analysed athletes had better balance than the controls. There were relationships between stature and body weight with postural stability in the case of canoeists. At the different measuring positions, a statistically significant asymmetries were observed in the athletes.

Our work in this study has some limitations like small sample size, lack of dynamic analysis of postural stability, tool used for body composition analysis which should be more accurate, like DXA which is the reference method, lack of the relationship of lateral asymmetry of parameters with the sport level and biomechanical/physical factors or conclusion done for only one gender (men). Nevertheless, we believe that our findings could be a worthwhile consideration about potentially connection between morphological somatic parameters and its side-to-side differentiation with postural stability in canoeists. Future studies should aim to develop the sport-specific level of morphofunctional asymmetry which would be optimal for sport results and health as well.

### Practical application

Due to the relationships found in present research between balance and certain somatic parameters with its side-to-side distribution, it is important to systematically monitor body composition and asymmetry among canoe athletes in view of their practical implications during training and in competitive settings. Further studies however are needed, with larger samples of athletes of both sexes and additional age categories to confirm our results.

## Supporting information

S1 Data (XLSX)

## References

[1] Stone MH, Stone M, Sands WA. Principles and practice of resistance training. Human Kinetics; 2007.

[2] Coldwells A, Atkinson G, Reilly T. The influence of skeletal muscle mass on dynamic muscle performance. World Wide Variation in Physical Fitness University of Levuen Levuen. 1993; 50–53.

[3] Teunissen JWA, Ter Welle SS, Platvoet SS, Faber I, Pion J, Lenoir M. Similarities and differences between sports subserving systematic talent transfer and development: The case of paddle sports. J Sci Med Sport. 2021;24: 200–205. doi: 10.1016/j.jsams.2020.09.005 32972845

[4] Rynkiewicz T, Rynkiewicz M. Zmienność wybranych uwarunkowań wyniku sportowego w kajakarstwie. AWF Poznań; 2012.

[5] Greve JMD, Cuğ M, Dülgeroğlu D, Brech GC, Alonso AC. Relationship between anthropometric factors, gender, and balance under unstable conditions in young adults. Biomed Res Int. 2013;2013: 850424. doi: 10.1155/2013/850424 23509788 PMC3581282

[6] Zdrodowska A, Wiszomirska I, Kaczmarczyk K, Kosmol A. Effects of anthropometric factors on postural stability in individuals with hearing impairment. Acta Bioeng Biomech. 2018;20: 109–115. 29658522

[7] Hamano S, Ochi E, Tsuchiya Y, Muramatsu E, Suzukawa K, Igawa S. Relationship between performance test and body composition/physical strength characteristic in sprint canoe and kayak paddlers. Open Access J Sports Med. 2015;6: 191–199. doi: 10.2147/OAJSM.S82295 26150737 PMC4480586

[8] Hagner-Derengowska M, Hagner W, Zubrzycki I, Krakowiak H, Słomko W, Dzierżanowski M, et al. Body structure and composition of canoeists and kayakers: analysis of junior and teenage polish national canoeing team. Biol Sport. 2014;31: 323–326. doi: 10.5604/20831862.1133937 25609891 PMC4296839

[9] Ackland TR, Ong KB, Kerr DA, Ridge B. Morphological characteristics of Olympic sprint canoe and kayak paddlers. J Sci Med Sport. 2003;6: 285–294. doi: 10.1016/s1440-2440(03)80022-1 14609145

[10] López-Plaza D, Alacid F, Muyor JM, López-Miñarro PÁ. Sprint kayaking and canoeing performance prediction based on the relationship between maturity status, anthropometry and physical fitness in young elite paddlers. J Sports Sci. 2017;35: 1083–1090. doi: 10.1080/02640414.2016.1210817 27433884

[11] Ducher G, Jaffré C, Arlettaz A, Benhamou C-L, Courteix D. Effects of long-term tennis playing on the muscle-bone relationship in the dominant and nondominant forearms. Can J Appl Physiol. 2005;30: 3–17. doi: 10.1139/h05-101 15855679

[12] Krzykała M, Leszczyński P. Asymmetry in body composition in female hockey players. Homo. 2015;66: 379–386. doi: 10.1016/j.jchb.2015.02.008 26077573

[13] Chen TL-W, Wong DW-C, Wang Y, Ren S, Yan F, Zhang M. Biomechanics of fencing sport: A scoping review. PLoS One. 2017;12: e0171578. doi: 10.1371/journal.pone.0171578 28187164 PMC5302478

[14] Rynkiewicz M, Starosta W. Asymmetry of paddling technique, its selected conditions and changeability in highly advanced kayakers. AWF Poznań and IASC Warszawa. 2011;35.

[15] Wietrzyński M, Mazur-Różycka J, Gajewski J, Michalski R, Różycki S, Buśko K. The assessment of muscle strength symmetry in kayakers and canoeists. Biomedical Human Kinetics. 2013;5: 65–71. doi: 10.2478/bhk-2013-0010

[16] Hawrylak A, Skolimowski T, Barczyk K, Bieć E. Asymetria w obrębie tułowia u osób trenujących wyczynowo różne dyscypliny sportu. Medycyna Sportowa. 2001;6: 232–235.

[17] Zakynthinaki MS, Milla JM, De Durana ALD, Martínez CAC, Romo GR, Quintana MS, et al. Rotated balance in humans due to repetitive rotational movement. Chaos. 2010;20: 013118. doi: 10.1063/1.3335460 20370273

[18] Ryew C-C, Lee A-R, Hyun S-H. Effect of muscle mass asymmetric between upper and lower limbs on the postural stability and shock attenuation during landing. J Exerc Rehabil. 2019;15: 488–492. doi: 10.12965/jer.1938188.094 31316946 PMC6614760

[19] Wilczyński J, Cieślik M, Maszczyk A, Zwierzchowska A. The Importance of Posture and Body Composition for The Stability and Selected Motor Abilities of Professional Handball Players. J Hum Kinet. 2022;82: 264–273. doi: 10.2478/hukin-2022-0025 36196344 PMC9465722

[20] Krzykała M, Karpowicz M, Strzelczyk R, Pluta B, Podciechowska K, Karpowicz K. Morphological asymmetry, sex and dominant somatotype among Polish youth. PLoS One. 2020;15: e0238706. doi: 10.1371/journal.pone.0238706 32915820 PMC7485883

[21] Lee EJ, Lee SA, Soh Y, Kim Y, Won CW, Chon J. Association between asymmetry in lower extremity lean mass and functional mobility in older adults living in the community: Results from the Korean Frailty and Aging Cohort Study. Medicine (Baltimore). 2019;98: e17882. doi: 10.1097/MD.0000000000017882 31702661 PMC6855585

[22] Kędziorek J, Błażkiewicz M, Kaczmarczyk K. Using nonlinear measures to evaluate postural control in healthy adults during bipedal standing on an unstable surface. Acta Bioeng Biomech. 2021;24. 38314480

[23] Andreoli A, Monteleone M, Van Loan M, Promenzio L, Tarantino U, De Lorenzo A. Effects of different sports on bone density and muscle mass in highly trained athletes. Med Sci Sports Exerc. 2001;33: 507–511. doi: 10.1097/00005768-200104000-00001 11283423

[24] Rynkiewicz M, Rynkiewicz T, Starosta W. Asymmetry of spinal segments mobility in canoeists and its relationship with racing speed. J Hum Kinet. 2013;36: 37–43. doi: 10.2478/hukin-2013-0004 23717353 PMC3661892

[25] Zemková E, Hamar D. The effect of task-oriented sensorimotor exercise on visual feedback control of body position and body balance. Hum Mov. 2010;11: 119–123.

[26] Davlin CD. Dynamic balance in high level athletes. Percept Mot Skills. 2004;98: 1171–1176. doi: 10.2466/pms.98.3c.1171-1176 15291203

[27] Nardone A, Schieppati M. The role of instrumental assessment of balance in clinical decision making. Eur J Phys Rehabil Med. 2010;46: 221–237. 20485225

[28] Kuczyński M, Podbielska M, Bieć D, Paluszak A, Kręcisz K. Podstawy oceny równowagi ciała: czyli co, w jaki sposób i dlaczego powinniśmy mierzyć? Acta Bio-Optica et Informatica Medica Inżynieria Biomedyczna. 2012;Vol. 18. http://yadda.icm.edu.pl/baztech/element/bwmeta1.element.baztech-fb26fd7b-7575-4986-b89b-dad2494461e1

[29] Arol P, Eroğlu KI. The effects of 8 week balance training on the kayaking performance of the beginners. Pedagogics, psychology, medical-biological problems of physical training and sports. 2018;22: 170–175. doi: 10.15561/18189172.2018.0401

[30] Bishop C, Turner A, Read P. Effects of inter-limb asymmetries on physical and sports performance: a systematic review. J Sports Sci. 2018;36: 1135–1144. doi: 10.1080/02640414.2017.1361894 28767317

[31] Vincent HK, Vincent KR. Rehabilitation and Prehabilitation for Upper Extremity in Throwing Sports: Emphasis on Lacrosse. Curr Sports Med Rep. 2019;18: 229–238. doi: 10.1249/JSR.0000000000000606 31385839

[32] Hewett TE, Di Stasi SL, Myer GD. Current concepts for injury prevention in athletes after anterior cruciate ligament reconstruction. Am J Sports Med. 2013;41: 216–224. doi: 10.1177/0363546512459638 23041233 PMC3592333

[33] Moreno LA, Rodríguez G, Guillén J, Rabanaque MJ, León JF, Ariño A. Anthropometric measurements in both sides of the body in the assessment of nutritional status in prepubertal children. Eur J Clin Nutr. 2002;56: 1208–1215. doi: 10.1038/sj.ejcn.1601493 12494306

[34] Impellizzeri FM, Rampinini E, Maffiuletti N, Marcora SM. A vertical jump force test for assessing bilateral strength asymmetry in athletes. Med Sci Sports Exerc. 2007;39: 2044–2050. doi: 10.1249/mss.0b013e31814fb55c 17986914

[35] Knapik JJ, Bauman CL, Jones BH, Harris JM, Vaughan L. Preseason strength and flexibility imbalances associated with athletic injuries in female collegiate athletes. Am J Sports Med. 1991;19: 76–81. doi: 10.1177/036354659101900113 2008935

[36] Papadatou-Pastou M, Ntolka E, Schmitz J, Martin M, Munafò MR, Ocklenburg S, et al. Human handedness: A meta-analysis. Psychol Bull. 2020;146: 481–524. doi: 10.1037/bul0000229 32237881

[37] Martin R. und Sailer, K.: Lehrbuch der Anthropologie, Band 1, Stuttgart, 1957. Gustav Fischer Verlag;

[38] Kushner RF, Schoeller DA, Fjeld CR, Danford L. Is the impedance index (ht2/R) significant in predicting total body water? Am J Clin Nutr. 1992;56: 835–839. doi: 10.1093/ajcn/56.5.835 1415001

[39] Demerath EW, Guo SS, Chumlea WC, Towne B, Roche AF, Siervogel RM. Comparison of percent body fat estimates using air displacement plethysmography and hydrodensitometry in adults and children. Int J Obes Relat Metab Disord. 2002;26: 389–397. doi: 10.1038/sj.ijo.0801898 11896495

[40] Brown SR, Brughelli M, Lenetsky S. Profiling Single-Leg Balance by Leg Preference and Position in Rugby Union Athletes. Motor Control. 2018;22: 183–198. doi: 10.1123/mc.2016-0062 28657806

[41] Arnold BL, Schmitz RJ. Examination of balance measures produced by the biodex stability system. J Athl Train. 1998;33: 323–327. 16558529 PMC1320582

[42] Dabbs NC, Sauls NM, Zayer A, Chander H. Balance Performance in Collegiate Athletes: A Comparison of Balance Error Scoring System Measures. Journal of Functional Morphology and Kinesiology. 2017;2: 26. doi: 10.3390/jfmk2030026

[43] Alacid F, Marfell-Jones MJ, Lopez-Minarro PA, Martinez I, Muyor JM. Morphological characteristics of young elite paddlers. 2011 [cited 19 Sep 2022]. https://repository.openpolytechnic.ac.nz/handle/11072/1955

[44] Bučinel AK, Supej M, Petrone N, Čuk I. How does body symmetry influence standing balance? Kinesiology. 2019;51: 52–59.

[45] Ridge BR, Broad E, Kerr DA, Ackland TR. Morphological characteristics of Olympic slalom canoe and kayak paddlers. European Journal of Sport Science. 2007;7: 107–113. doi: 10.1080/17461390701478357 14609145

[46] Robinson MG, Holt LE, Pelham TW. The technology of sprint racing canoe and kayak hull and paddle designs. International Sports Journal. 2002;6: 68–85.

[47] Stambolieva K, Diafas V, Bachev V, Christova L, Gatev P. Postural stability of canoeing and kayaking young male athletes during quiet stance. Eur J Appl Physiol. 2012;112: 1807–1815. doi: 10.1007/s00421-011-2151-5 21909987

[48] Carter JL, Ross WD, Aubry SP, Hebbelinck M, Borms J. 4. Anthropometry of Montreal Olympic Athletes. Physical structure of olympic athletes. Karger Publishers; 1982. pp. 25–52.

[49] Dimakopoulou E, Blazevich AJ, Kaloupsis S, Diafas V, Bachev V. Prediction of stroking characteristics of elite rowers from anthropometrics variables. Serbian J Sport Sci. 2007;1: 91–97.

[50] Alonso AC, Luna NMS, Mochizuki L, Barbieri F, Santos S, Greve JMD. The influence of anthropometric factors on postural balance: the relationship between body composition and posturographic measurements in young adults. Clinics (Sao Paulo). 2012;67: 1433–1441. doi: 10.6061/clinics/2012(12)14 23295598 PMC3521807

[51] Kejonen P, Kauranen K, Vanharanta H. The relationship between anthropometric factors and body-balancing movements in postural balance. Arch Phys Med Rehabil. 2003;84: 17–22. doi: 10.1053/apmr.2003.50058 12589615

[52] Hue O, Simoneau M, Marcotte J, Berrigan F, Doré J, Marceau P, et al. Body weight is a strong predictor of postural stability. Gait Posture. 2007;26: 32–38. doi: 10.1016/j.gaitpost.2006.07.005 16931018

[53] Kameyama O, Shibano K, Kawakita H, Ogawa R, Kumamoto M. Medical check of competitive canoeists. J Orthop Sci. 1999;4: 243–249. doi: 10.1007/s007760050099 10436270

[54] McKeon PO, Hertel J. Systematic review of postural control and lateral ankle instability, part I: can deficits be detected with instrumented testing. J Athl Train. 2008;43: 293–304. doi: 10.4085/1062-6050-43.3.293 18523566 PMC2386423

[55] Keller JL, Housh TJ, Smith CM, Hill EC, Schmidt RJ, Johnson GO. Sex-Related Differences in the Accuracy of Estimating Target Force Using Percentages of Maximal Voluntary Isometric Contractions vs. Ratings of Perceived Exertion During Isometric Muscle Actions. J Strength Cond Res. 2018;32: 3294–3300. doi: 10.1519/JSC.0000000000002210 29176386

[56] Rynkiewicz M, Baumgarten M, Mroczkowski A. Does paddling in canoes cause asymmetry of muscle mass and fat mass in high level athletes? Rocznik Lubuski. 2018;44: 355–366.

[57] Maloney SJ. The Relationship Between Asymmetry and Athletic Performance: A Critical Review. J Strength Cond Res. 2019;33: 2579–2593. doi: 10.1519/JSC.0000000000002608 29742749

[58] Virgile A, Bishop C. A Narrative Review of Limb Dominance: Task Specificity and the Importance of Fitness Testing. J Strength Cond Res. 2021;35: 846–858. doi: 10.1519/JSC.0000000000003851 33470600

[59] Afonso J, Peña J, Sá M, Virgile A, García-de-Alcaraz A, Bishop C. Why sports should embrace bilateral asymmetry: a narrative review. Symmetry. 2022;14: 1993.

[60] Zemková E. Assessment Of Balance In Sport: Science And Reality. Serb J Sports Sci. 2011.

